# Phenols content and 2-D electrophoresis protein pattern: a promising tool to monitor *Posidonia *meadows health state

**DOI:** 10.1186/1472-6785-7-6

**Published:** 2007-07-30

**Authors:** Luciana Migliore, Alice Rotini, Davide Randazzo, Nadia N Albanese, Agata Giallongo

**Affiliations:** 1 Università "Tor Vergata", Dipt. Biologia, Via della Ricerca Scientifica, I-00133 Roma, Italy; 2 Università di Palermo, Dipt. Oncologia Sperimentale e Applicazioni Cliniche, 90146 Palermo, Italy; 3 Istituto di Biomedicina e Immunologia Molecolare, Consiglio Nazionale delle Ricerche, 90146 Palermo, Italy

## Abstract

**Background:**

The endemic seagrass *Posidonia oceanica *(L.) Delile colonizes soft bottoms producing highly productive meadows that play a crucial role in coastal ecosystems dynamics. Human activities and natural events are responsible for a widespread meadows regression; to date the identification of "diagnostic" tools to monitor conservation status is a critical issue. In this study the feasibility of a novel tool to evaluate ecological impacts on *Posidonia *meadows has been tested. Quantification of a putative stress indicator, *i.e*. phenols content, has been coupled to 2-D electrophoretic protein analysis of rhizome samples.

**Results:**

The overall expression pattern from *Posidonia *rhizome was determined using a preliminary proteomic approach, 437 protein spots were characterized by p*I *and molecular weight. We found that protein expression differs in samples belonging to sites with high or low phenols: 22 unique protein spots are peculiar of "low phenols" and 27 other spots characterize "high phenols" samples.

**Conclusion:**

*Posidonia *showed phenols variations within the meadow, that probably reflect the heterogeneity of environmental pressures. In addition, comparison of the 2-D electrophoresis patterns allowed to highlight qualitative protein expression differences in response to these pressures. These differences may account for changes in metabolic/physiological pathways as adaptation to stress. A combined approach, based on phenols content determination and 2-D electrophoresis protein pattern, seems a promising tool to monitor *Posidonia *meadows health state.

## Background

**Figure 1:**
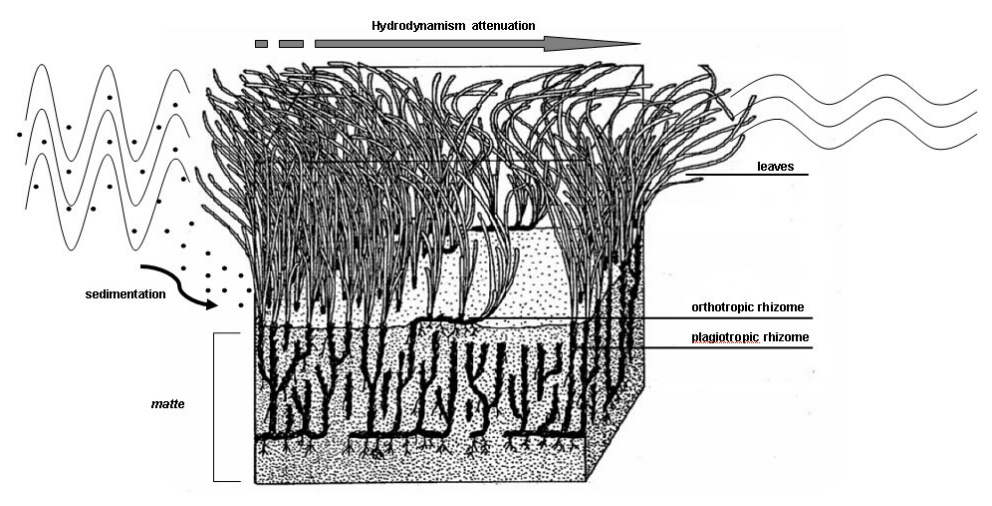
Schematic representation of *Posidonia *meadow (*matte*, rhizomes and leaves) and its effect on sediment stabilization and reduction of hydrodynamism (modified from Boudouresque and Meinesz [55]).

*Posidonia oceanica *(L.) Delile (fig. 1) is a Mediterranean endemism. Plants colonize soft bottoms producing large meadows that span from the sea surface to 35–40 m depth. Meadows are highly productive ecosystems, as they produce high amount of oxygen and organic compounds, sustain complex food nets, act as a nursery/refuge for several species. They also play a crucial role in coastal preservation, by stabilizing sediments and reducing hydrodynamics effects (see fig. 1) [1,2].

Many human activities and natural events are responsible for the widespread meadows regression, such as modified hydrogeological regime and littoral transport [3-6], pollution [7-11], aquaculture [12-15], trawling [16,17], anchorages [18-21], placing of cable/pipes or damping [22,23], in addition to grazing, sea storms, climatic changes, etc. [24-26]. All lead to alterations of *Posidonia *ecosystems.

Phenolic compounds are widespread secondary metabolites in plants. They play a role in herbivore/pathogen protection [27,28] and are considered stress indicators in terrestrial plants [29-38]. Phenols compounds have been identified in marine phanerogames [39-41] and high concentrations of phenolic compounds in *Posidonia *leaves were found in a few cases a) under competition with *Caulerpa taxifolia *[42,43], b) under mercury contamination [44], c) nearby offshore aquaculture cages [45]. An attempt to identify specific phenolic compounds in *Posidonia *leaves in response to different environmental pressures did not give clear-cut results [46]. This may be due to the fact that *Posidonia *leaves are temporary structures with a relatively short lifespan (about 7 months) at the Mediterranean mid-latitudes. Therefore they show marked seasonal fluctuations in common physiological processes – including synthesis and accumulation of phenolic compounds [47]. Rhizomes have a lifespan much longer than leaves: consequently, they undergo less marked fluctuations and may carry the memory of experienced environmental pressures.

The 2-D electrophoresis protein analysis produces maps of all the expressed proteins, in a given time and under a specific environmental condition. The protein pattern is a dynamic entity varying from cell to cell in the same organism, it is constantly modulated by external and internal signalling and reflects changes in the physiological state. The proteomic approach, based on the simultaneous separation of hundreds of proteins in the same 2D-electrophoretic gel, represents a powerful tool to monitor the "health state" of ecosystems, by comparing quantitative/qualitative pattern differences of protein expression in organisms living in polluted/non-polluted areas.

In this work we choose the rhizome, in particular the basal section, as the most reliable plant portion to evaluate possible alterations of both phenols content and protein expression.

The aim of this study was to verify the feasibility of phenols quantification coupled to 2-D electrophoretic protein analysis in rhizomes, as a novel "diagnostic" tool to monitor *Posidonia *meadows conservation status.

## Results and discussion

### Phenols content

Total phenols were measured in distal, intermediate and basal sections of sampled rhizomes. Mean value (mg/g fresh weight) of the distal sections was 25.6 (n = 60; SE 1.1) ranging from 18.9 to 35, intermediate sections mean was 25.1 (n = 60; SE 1.1) ranging from 15.3 to 36.7 and basal sections mean was 23.7 (n = 60; SE 1.4) from 7.1 to 35.2.

**Figure 2:**
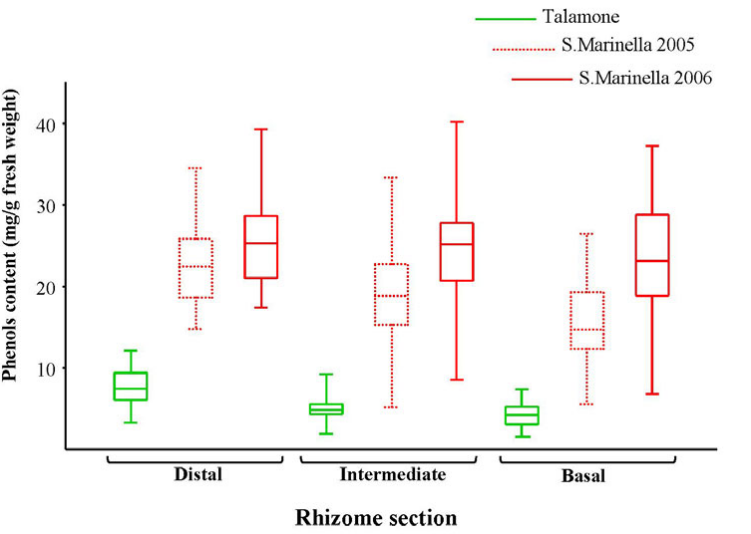
**Distribution of phenols content in rhizome distal, intermediate and basal sections**. Phenols values (mg/g fresh weight) are represented as *box-plots*: the box contains 50% data (the extremes of that box are the Q1 and Q3, 1^st ^and 3^rd ^quartiles), the internal horizontal segments represent median of the distributions (Q2 value, 2^nd ^quartile), 'whiskers' range from the lowest to the highest value. The box plots from S. Marinella meadow samples collected in 2006 (red, solid line) and 2005 (red, dotted line), and from Talamone meadow samples (green) are reported.

Data of total phenols content in samples collected in 2006 from the S. Marinella meadow were compared to data previously obtained with identical experimental procedure from rhizomes of the same meadow collected in 2005 and from rhizomes of the Talamone meadow (Grosseto, Italy, 2002). Samples from the well preserved meadow of Talamone [54] showed the lowest and less scattered phenols values, samples from the S. Marinella meadow (year 2006) showed highly scattered values (Fig. 2). We found that the overall phenols content in S. Marinella-2005 samples was lower than the content detected in 2006 samples, however in both cases values were higher than the ones obtained from the Talamone samples (compare box-plots in Fig.2).

Phenols content differences between Talamone and S. Marinella-2006 samples are statistically significant for all the sections (Mann-Whitney, p << 0.001), differences between S. Marinella-2005 and S. Marinella-2006 samples are highly significant for basal and intermediate sections (Mann-Whitney, p << 0.001), significant for the distal section (Mann-Whitney, p = 0.00146). The lowest phenols content may be consistently associated with the good health state of the Talamone meadow [53].

In this study on the S. Marinella meadow the three lowest phenols contents found in rhizome basal sections were: 7.1 mg/g ± 0.5, 14.4 mg/g ± 0.2 and 14.5 mg/g ± 0.8; the three highest were: 28.9 mg/g ± 0.2, 33.6 mg/g ± 2.0 and 35.2 mg/g ± 2.8. *Posidonia *shoots from these six sampling sites were chosen for protein analyses.

### Protein analysis

In order to assess a possible match between different phenols content and variations in protein expression, at first we determined the overall expression pattern of *Posidonia *rhizome by 2-D electrophoresis. The polypeptides falling within the experimental window of p*I *3–9 and 12–200 kDa and sufficiently abundant to be detected by the silver staining procedure were taken into account. Protein patterns from low and high phenols samples were combined and the experimental values of p*I *and molecular weight for each isoelectric spot were calculated by a dedicated computer software using reference proteins with known p*I *and molecular weight, commonly called "anchors" (see Materials and Methods section). Proteins, accounting for a total of 437 spots, ranged from p*I *5.11 to p*I *8.66 with an apparent molecular weight ranging from 13306 Da to 95563 Da (see additional file 1).

**Figure 3:**
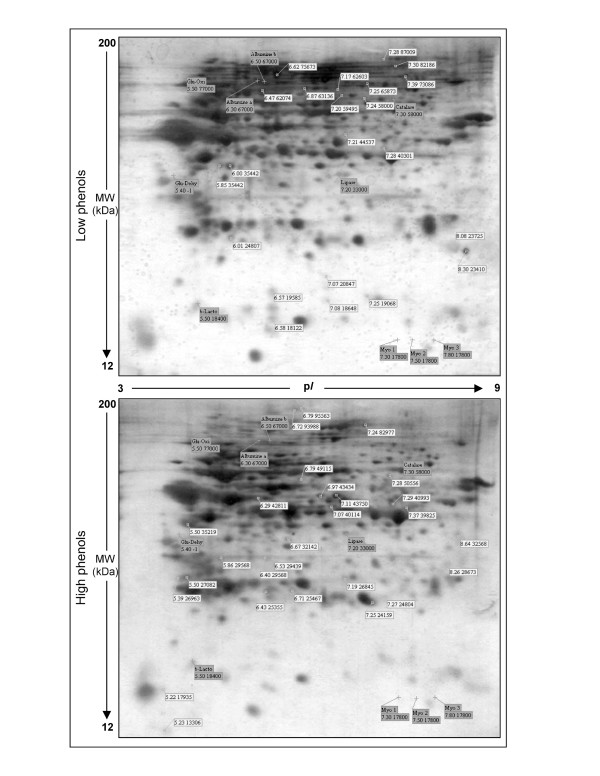
**Representative 2-DE patterns of *Posidonia *rhizome proteins**. *Upper panel*, proteins isolated from low phenols samples. *Lower panel*, proteins isolated from high phenols samples. About 15 *μ*g of proteins from dry powder of *Posidonia *rhizome were separated on IPG gel strip (7 cm, 3–10 NL) followed by SDS-PAGE on a vertical mini-gel (12%T). Peculiar protein spots are labelled, p*I *and molecular weight values are indicated (white background). Positions of protein markers are indicated and labelled with name and p*I*/molecular weight values (gray background). Protein markers (in alphabetic order) are: Albumine a; Albumine b; Catalase; Glucose-1-Dehydronase (Glu-Dehy); Glucose oxidase (Glu-Oxi); *β*-Lactoglobuline (b-Lacto); Myoglobine subunits (Myo 1, Myo 2, Myo 3). Numbers on the left refer to the position of the molecular weight standards and numbers in the middle indicate the p*I *range.

Two representative 2-D gels from low and high phenols are shown in Fig. 3. Computer-assisted cross-comparison revealed qualitative differences that accounted for differentially expressed proteins: 22 spots are peculiar of low phenols whereas 27 spots characterize high phenols samples, accounting for 5.0 and 6.4% of the entire map, respectively. These differences were consistently found in all the examined samples.

At low phenols content, differentially expressed proteins ranged from p*I *5.85 to p*I *8.30, with an apparent molecular weight ranging from 18122 Da to 87009 Da. In high phenols samples, isoelectric point of differentially expressed proteins was comprised between p*I *5.22 and p*I *8.64, with an apparent molecular weight from 13306 Da to 95563 Da (Table 1).

**Table 1:** Peculiar protein spots identified in the 2-D electrophoretic maps of low phenols (22 spots) and high phenols (27 spots) *Posidonia *rhizome samples

**Low phenols**		**High phenols**	
p*I*	*MW (Da)*	p*I*	*MW (Da)*

5.85	35442	5.22	17935
6.00	35442	5.23	13306
6.01	24807	5.39	26963
6.47	62074	5.50	35219
6.57	19585	5.50	27082
6.58	18122	5.50	28926
6.62	75673	5.86	29568
6.87	63136	6.29	42811
7.07	20847	6.40	29568
7.08	18648	6.43	25355
7.17	62603	6.53	29439
7.20	59495	6.67	32142
7.21	44537	6.71	25467
7.24	58000	6.72	93988
7.25	65873	6.79	95563
7.25	19068	6.79	49115
7.28	87009	6.97	43434
7.28	40301	7.07	40114
7.30	82186	7.11	43750
7.39	73086	7.19	26845
8.08	23725	7.24	82977
8.30	23410	7.25	24159
		7.27	24804
		7.28	50556
		7.29	40993
		7.37	39825
		8.26	28673
		8.64	32568

The experimental values of p*I *and molecular weight (MW) for every isoelectric spot were calculated with ImageMaster 2D Platinum System.

Although an identity was not assigned to the differentially expressed polypeptides, they have been firstly characterized by assigning a molecular mass and a total charge.

## Conclusion

It has been suggested that phenols content is an indicative trait of environmental stress in *Posidonia oceanica *[42-47,53]. In this work we choose the rhizome basal section as the most reliable material to measure this putative marker of ecosystem imbalance. The *Posidonia *shoots utilized in this study showed that variations in the phenols content exists within the examined meadow (S. Marinella, Italy), probably reflecting environmental pressures heterogeneity. Moreover, comparison of phenols content in the same meadow at one year time distance showed an increase in the overall values.

We have constructed the first 2-D electrophoretic map of *Posidonia oceanica *rhizome, made of 437 protein spots, characterized by p*I *and molecular weight.

Usually, the bi-dimensional protein pattern is typical of the physiological state and varies under different environmental conditions. Thus, the patterns comparison allows the highlighting of protein expression differences in response to stresses. Here we showed, by comparison of samples belonging to sites with low or high phenols content, that certain protein spots present in "low phenols" are absent in "high phenols" and *vice versa*. This may account for changes in metabolic/physiological pathways as adaptation to stress, including activation/repression of coordinate sets of genes. Differences cover the 5–6% of the entire protein map and match the plants phenols response. To the best of our knowledge, this is the first attempt to correlate the pattern of expressed proteins to a putative stress indicator, such as phenols content, in *Posidonia *rhizome.

Although the identification of each protein spot needs further investigation and might be hampered by the lack of extensive database information, the combined approach, based on phenols content determination and 2-D electrophoresis protein pattern, seems a promising tool to monitor *Posidonia *meadows health state.

## Methods

### Sampling, conservation and sample selection

*Posidonia oceanica *was sampled from the Santa Marinella meadow (Rome, Italy), Site of Community Importance (according to Habitat Directive 92/43/EEC), spanning from Capo Linaro to Santa Severa, for a 13.5 km coastline and covering a surface of 1,800 ha.

Shoots were sampled in May 2006 in 20 randomly chosen sites (depths from 7.5 to 13.5 m). At least 4 orthotropic shoots per sampling site were collected and maintained at 4°C in the dark until the arrival to the laboratory. Plants were first rinsed in 0.1 Triton-X (Sigma) and then in distilled water to remove epiphytes and contaminants.

At least 3 shoots per site were stored at -20°C until processing for phenol analysis, and at least 1 at -80°C for protein analyses. Total phenols concentration was determined in duplicate on three different rhizomes for sampling site according to Folin-Ciocalteau procedure [48], each shoot was dissected into three sections (about 1/3 of the total length): basal, intermediate and distal; about 125 mg fresh weight of each section were separately processed. Once established the sampling sites in which the three highest and lowest phenols values were found, shoots from these sampling sites were selected for protein analysis.

### Protein extraction and electrophoresis

Protein were extracted according to the Wang et al. [49] protocol originally developed for recalcitrant plant tissues (leaves and flesh). This protocol, previously utilized for *Posidonia *leaves [50], was modified for *Posidonia *rhizomes.

Briefly, frozen rhizomes were peeled off to remove all cortical tissues, and a piece of the basal section (250 mg) was ground in liquid N_2 _using a mortar with pestle. The powdered tissue was subjected to phenols extraction in the presence of SDS, as described [49].

The protein pellet was dried and dissolved in 2-DE rehydration solution [8 M urea, 2 M thiourea, 2% CHAPS, 50 mM dithiothreitol (DTT), 0.2% carrier ampholytes (3–10 Bio-Lyte Ampholyte, Bio-Rad Laboratories)], supplemented with proteases and phosphatases inhibitors (Sigma). Protein content was measured by Bradford protein assay (Bio-Rad Laboratories) using Bovine Serum Albumine as standard.

Protein samples (15 *μ*g) were applied in 155 μl of 2-DE rehydration solution to 7 cm Readystrip IPG (*Immobilized pH Gradient*, pH 3–10 NL, Bio-Rad Laboratories), by incubating overnight. The isoelectrofocusing (IEF) was performed at room temperature using the ZOOM IPG Runner™ Mini-cell (Invitrogen), applying 500 V for 4 hrs (1 mA/strip; 0.5 W/strip).

Focused strips were equilibrated using DTT and iodoacetamide solutions, positioned on a 12% acrylamide SDS-PAGE minigel 1 mm thick [51], according to standard procedures. After electrophoresis, resolved proteins were visualized by acidic silver staining that allows to detect as low as 0.5–1 ng of protein per spot [52]. Proteome p*I *markers were from SERVA Electrophoresis (Heidelberg). Each protein sample was subjected at least to 2 parallel runs of isolectrofocusing and second dimension electrophoretic separation to assess proteomic pattern reproducibility.

### Image processing and data analysis

Silver-stained gels were digitised using a Trust EasyConnect 19200 scanner, generating 2.4 Mb images. The images were saved as *Tiff format *and imported into the ImageMaster 2-D Platinum software (Amersham, version 6.0). Spot selection was performed using default selection parameters [53]. For the attribution of isoelectric points and relative molecular masses we utilized as internal standard a mixture of 10 protein with known identities (see Fig. 3 and related legend).

## Authors' contributions

LM and AG conceived and designed the study. AR sampled plants. AR and DR carried out the laboratory analyses, and analysed the data. NNA processed 2-DE images. LM and AG wrote the manuscript with help from AR and DR. All authors read and approved the final manuscript.

## Supplementary Material

Additional file 1 List of the 437 protein spots identified from *Posidonia *rhizome. The experimental values of p*I *and molecular weight (MW) for every isoelectric spot were calculated with ImageMaster 2D Platinum System. The percent of each spot with respect to the total spots volume is also reported. Click here for file
